# Supplementary material for: Model-Agnostic Neural Mean Field With The Refractory SoftPlus Transfer Function
Source: bioRxiv. 2024 Feb 6:2024.02.05.579047. Preprint. [Version 1] doi: 10.1101/2024.02.05.579047 (PMC10871173; doi:10.1101/2024.02.05.579047)
Supplement: Supplement 1 [file NIHPP2024.02.05.579047v1-supplement-1.pdf]

bioRxiv preprint doi: https://doi.org/10.1101/2024.02.05.579047; this version posted February 6, 2024. The copyright holder for this preprint (which was not certified by peer review) is the author/funder, who has granted bioRxiv a license to display the preprint in perpetuity. It is made available under aCC-BY 4.0 International license.

Supplementary Information

A Analytical Transfer Function

The mean inter-spike interval (ISI) for an LIF neuron under the diffusion approximation has been known for a long time [29]. It has a complicated functional form, even after significant simplification using the assumptions that the resting and reset potential of the neuron are identical and that the input has zero mean. Here erfi is the imaginary error function, and the notation !! refers to the double factorial:

$$M(R) = T_{\text{ref}} + \frac{\tau}{2}\left(\sum_{n=1}^{\infty} \frac{y^{2n}}{n(2n-1)!!} + \pi \operatorname{erfi}\left(\frac{y}{\sqrt{2}}\right)\right)$$

$$\text{where } y = (V_{\text{thresh}} - V_{\text{rest}})\sqrt{\frac{2}{\tau R q^2}}$$

For the LIF neuron, the transfer function is the reciprocal $M(R)^{-1}$. If $T_{\text{ref}} = 0$, the transfer function is a smooth curve which is near zero at low R but asymptotically grows as $R^{1/2}$, as shown in figure S1. Also shown in the figure is a neuron with a nonzero absolute refractory period, resulting in a transfer function which grows more slowly with R, towards an asymptotic maximum firing rate $1/T_{\text{ref}}$.

The neuron was numerically simulated at 500 different input rates r. When q varies for a fixed r, the total number N of presynaptic neurons (yielding total input rate $R = Nr$) is changed to hold D constant so that all conditions are equivalent according to the diffusion limit. Since Poisson processes superimpose linearly, this is only a visual convenience which allows plotting firing rates for different q against the same horizontal axis. Firing rates were then calculated by dividing the total number of spiking events by the total simulation time.

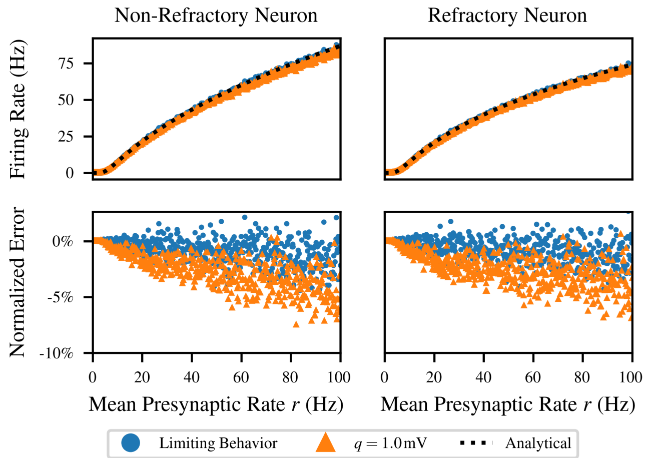

Figure S1: Comparison of the theoretical transfer function $M(R)^{-1}$ to simulated data for an LIF neuron with $\tau = 10\,\text{ms}$, $V_{\text{thresh}} = -50\,\text{mV}$, $V_{\text{rest}} = -65\,\text{mV}$, and refractory period T_{ref} either zero (left) or $2\,\text{ms}$ (right). The diffusion limit is represented by a simulation with a small PSP amplitude and large number of presynaptic neurons $q = 0.1\,\text{mV}$ and $N = 100000$ (blue). However, violating the diffusion approximation by changing the PSP to $q = 1\,\text{mV}$ and $N = 1000$ (orange) leads to significant deviation from the analytical solution (dashed line).

bioRxiv preprint doi: https://doi.org/10.1101/2024.02.05.579047; this version posted February 6, 2024. The copyright holder for this preprint (which was not certified by peer review) is the author/funder, who has granted bioRxiv a license to display the preprint in perpetuity. It is made available under aCC-BY 4.0 International license.

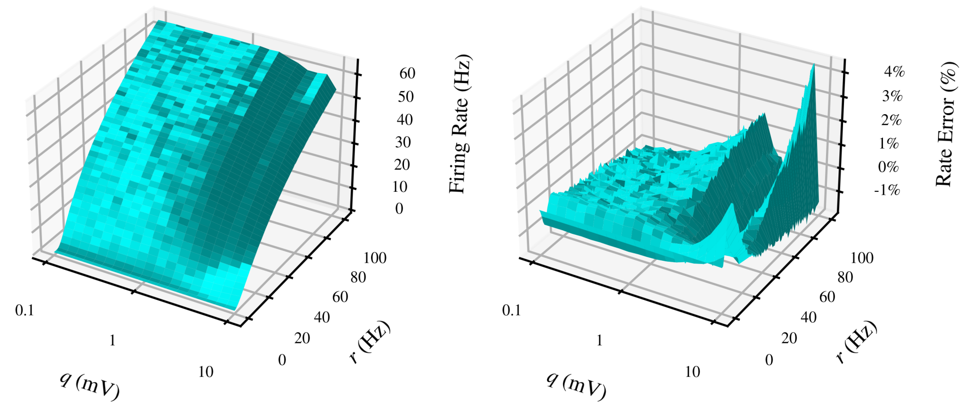

Figure S2: The transfer function as a two-dimensional function of PSP amplitude q and presynaptic rate r (left), and the error landscape of a single PSP-dependent Refractory SoftPlus fit to this data (right).

Although the analytical transfer function is accurate in the diffusion limit, the simulation in figure S1 also demonstrates a case where the analytical diffusion approximation breaks down. The limiting behavior is represented by a simulation with a sufficiently small PSP amplitude $q = 0.1\,\mathrm{mV}$. Increasing the PSP to the biologically reasonable value of $q = 1\,\mathrm{mV}$ (orange) leads to significant deviation from the analytical solution. Similar deviations also occur due to the use of a finite simulation timestep, which leads to a small error even in the better of the two curves in figure S1, as well as to compounded errors from both of these sources in figure 2.

B Varying Postsynaptic Potentials

Theoretically, the use of q in equation (7) is redundant, as it is equivalent to rescaling β and σ_0, but because it is derived from the diffusion coefficient D, it acts as a first-order approximation to the effect of PSP amplitude. The only effect of this is to make fitting numerically easier, as the parameters vary less with q. However, it is possible to directly model this dependence to obtain a transfer function with better performance across multiple values of q. We observed empirically that q has relatively little influence on the fitted values σ_0 and T_{ref}, so we modeled α and β as having a first-order dependence on q with a shared parameter γ:

$$\alpha(q) = (1 + \gamma q)\alpha_0$$
$$\beta(q) = (1 + \gamma q)\beta_0$$

This substitution yields the PSP-dependent Refractory SoftPlus transfer function:

$$S_{\mathrm{ref},q}(R; \alpha_0, \beta_0, \gamma, \sigma_0) = \left(T_{\mathrm{ref}} + \alpha(q)\,\mathrm{SoftPlus}(q\sqrt{R} - \sigma_0\ ;\ \beta(q))^{-1}\right)^{-1}$$

If the simulation data used to fit the transfer function includes varying values of q, this version of the function can be fitted instead, which achieves significantly better results using only one additional parameter γ. This approach is shown in figure S2, where a simulated transfer function was calculated for LIF neurons subject to Poisson

bioRxiv preprint doi: https://doi.org/10.1101/2024.02.05.579047; this version posted February 6, 2024. The copyright holder for this preprint (which was not certified by peer review) is the author/funder, who has granted bioRxiv a license to display the preprint in perpetuity. It is made available under aCC-BY 4.0 International license.

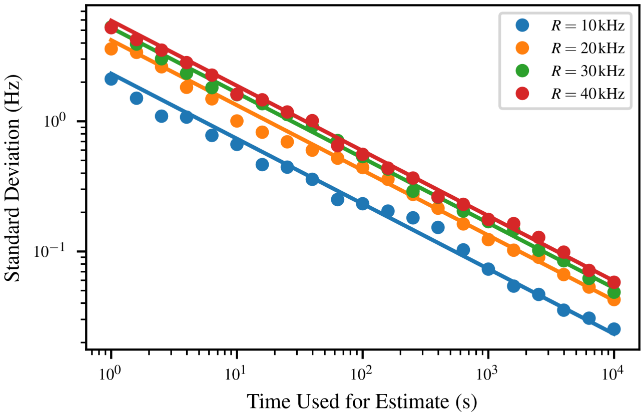

Figure S3: Variance in the firing rate estimate between multiple simulations (colored points) as a function of the simulation time T, compared to the approximation (solid lines) for a few different firing rates.

input under a range of q and r, and a single PSP-dependent Refractory Softplus fit was performed.

We observe an overall RMS error on par with the simulations of the single neuron, which is able to account for the decrease in firing rate for large q. As in figure S1, N was covaried with q as to keep the range of $D = q^2 N r$ (equation 3) the same. This choice was made for visualization purposes; fits with a broader range of input R and D due to unmatched N achieved similar performance.

C Variance in Firing Rate Estimates

In this section, we provide some context for the magnitude of the error displayed in figure 4 for the convergence of fitted SoftPlus curves as a function of the amount T of simulation time available. We do this by comparing the error between the fitted curves and the final long-time simulation run with the error in the firing rate estimates generated from short-time simulations.

If the neuron fires according to a Poisson process with rate r over a time interval T, the expected number of firings in the time interval is rT, with variance rT. Therefore, the variance of an estimate of the neuron's firing rate can be approximated as r/T.

We can derive the same result by imagining using bootstrapping to calculate the variance of a binary spike raster. If the neuron fired exactly rT times within the time T, which is broken up into bins of a very small duration h, then each bootstrap fold is T/h samples of a Bernoulli random variable with rate rh. The sum of these variables follows a binomial distribution with mean rT and variance $rT(1 - rh) \approx rT$, so this firing rate estimate also has mean r and variance r/T. Note that this method assumes that the bins of the raster are independent, which is equivalent to the Poisson assumption.

Figure S3 depicts the standard deviation of 50 firing rate estimates of single LIF neurons with four different input rates. The horizontal axis is the length of the simulation used to calculate the neuron's firing rate, as in figure 4. Even though the neurons are not actually Poisson, the approximation appears quite accurate for the case considered.

As in figure 4, a full simulation of $T = 10^4\,\mathrm{s}$ was performed, and data restriction

bioRxiv preprint doi: https://doi.org/10.1101/2024.02.05.579047; this version posted February 6, 2024. The copyright holder for this preprint (which was not certified by peer review) is the author/funder, who has granted bioRxiv a license to display the preprint in perpetuity. It is made available under aCC-BY 4.0 International license.

was simulated by restricting attention only to the beginning of this interval. As an artifact of this, adjacent points are correlated in their deviation from the approximation, which we expect would not happen if independent simulations were employed.

In figure 4, the displayed values are the normalized RMS error across an entire empirical firing rate curve. This approximation can be employed to describe the behavior of the error number in the figure, given the true underlying population value of the full sequence $\{r_i\}$ of firing rates of M different neurons in the reported population. The expected squared error of each estimate is its variance r_i/T, so the normalized RMS error ε of the entire empirical firing rate curve is given by:

$$\varepsilon = \frac{1}{\max_i r_i} \sqrt{\frac{1}{M} \sum_{i=1}^{M} \frac{r_i}{T}} \tag{10}$$

Although the precise value now depends on a significant number of variables which are difficult to know *a priori*, it is at least clear that the expected RMS error will scale as $T^{-1/2}$ just like the standard deviation of a population of single-neuron estimates.
